# Materia Medica and Therapeutics

**Published:** 1858-05

**Authors:** 


### MATERIA MEDICA AND THERAPEUTICS.

ON THE MEDICINAL ACTION OF LUPULINE. By *Walter Jauncey, L.R.C.S. Ed.*—The hop, although known long since as a medicinal plant, has not been generally used as such, unless in combination; and most authors on materia medica mention it as deserving of attention.

Two years ago, being in a hop district at the period of gathering hops, I thought it a favorable opportunity for commencing an inquiry into their medicinal efficacy. It might not, however, be out of place, to give first a short sketch of the mode of cultivation and preparation of the hop of commerce, as observed in the Worcester and Hereford plantations. The hops are planted about March, and growing but a little the first year, bear scantily the next, and come to their full vigor in the third or fourth. In May, poles are stuck in the ground for the young vines to grow upon. The plants are arranged either on the summit of parallel ridges, (so that aisles are formed when the vines have twined to the tops of the poles,) or in clumps at right angles to each other on the level. Flowering in July, the female plants bring their catkins or hops to maturity in September. These are picked from the vines by hand in the hopyards, and are brought to kilns, where they are spread upon horse-hair cloths, and dried by coke fires, and often are bleached by means of sulphurous acid vapor. This practice is, however, I believe, illegal. The dried hops are then broken, by means of ordinary rollers and sieves, or by machines for the purpose, and are lastly pressed into bags or pockets, and constitute the hops of commerce.

The virtue of the hop, as is well known, is concentrated in the yellow powder at the base of the scales called lupuline. In the process of breaking the hops, mentioned above, a large proportion of this is knocked out, some being lost, and some is mixed afterwards with the broken hops, so that the proportion contained in different samples of even good hops, is very variable. I have found it range in my experiments from one-fifth to one-ninth. This shows, that for medicinal purposes, it is necessary that the lupuline, and not the hops, should be used; and it is worthy of note that hops, unless broken in the injurious way described, do not sell so well in the market.

The lupuline so obtained is a powder of a deep-yellow color, sticking when taken between the fingers, gradually sinks in water, and possesses a peculiar, not unpleasant odor, and a bitterish aromatic taste: mixed with the lupuline are fragments of the broken strobiles and some of the hop seeds. Lupuline loses activity by exposure to air, its color is spoiled, and its efficacy impaired by access of light. I have noticed that it is necessary to exhibit larger doses of old than fresh lupuline. The most important constituent of lupuline appears to be a volatile oil, which is partially soluble in water, and very soluble in alcohol and ether; its smell resembles, but is stronger and less pleasant than that of hops, and there is an unpleasant acridity in the taste. Some writers have described the color as yellowish, but I have always obtained it of a mahogany hue, although I have taken care that it should not be empyreumatized. The partial solubility of the oil in water renders it troublesome to distil, since, if you use much water, you break the retort. After having, in spite of various expedients which I adopted, broken several glass retorts, I used a tin one, which enabled me to obtain the desired result, though a copper one would have been preferable to tin. A striking peculiarity about the taste of lupuline is its evanescence; and this is important, as it enables one to distinguish the hop from other bitters substituted for it, there being, as Dr. Christison remarks in the *Edinburgh Monthly Journal* for 1853, but one other plant, a leguminous one, in New South Wales, which resembles it in this particular; but when the pure volatile oil is placed on the tongue, an unpleasant flavor, somewhat similar to that caused by inhalation of prussic acid, lingers about the pharynx for some time. The oil of hop is, I feel confident, formed during the process of drying, as I have failed to obtain it from the ripe undried hop; and a tincture of the undried hop, though decidedly bitter, lacks the aromatic flavor found in the tincture of the dried lupuline. For investigating this point, I was obliged to use the entire hop, in consequence of the impossibility of separating the lupuline from the scales of the undried hop. In making the tincture, however, I used the undried hop, in the proportion of six to one of the lupuline. I should observe, that the oil, by keeping, becomes resinified, and that the resin is of a yellowish color, different from that of the oil, when fresh distilled.

From the observations I have made on the action of the oil, I conclude that it is sedative and anodyne. It relieves pain without necessarily producing sleep. Large doses of lupuline reduce the frequency of the pulse from 20 to 30 beats in a minute, (and Dr. Maton lowered the pulse even 36 beats in the minute,) and finally bring on pain in the head, nausea, and loss of appetite. These effects are produced by the oil, whether inhaled or swallowed. In full doses it acts also somewhat as a diuretic; and I have seen good evidence of its power in diminishing and allaying the venereal appetite. On one occasion I took repeated doses of the lupuline for six hours, 10 grains every half-hour, or 120 grains in all, when the pulse became lower by 30 beats in the minute, and intermittent. This was accompanied by such an uncomfortable sensation of faintness, that I did not carry the experiment further. I made the trial with every care, at a period when I was free from disturbance. On another day, about 2 P.M., I inclosed myself in a room while lupuline was being distilled in an open retort. The peculiar smell of the oil soon pervaded the room. My pulse was then 84, and I felt quite well and comfortable; in half an hour my pulse was reduced 15 beats, and I had a violent pain in the head. I continued the inhalation another half-hour, when the pulse had fallen to 60, and was occasionally intermittent; the headache became insupportable, and I felt an inclination to vomit. I discontinued the inhalation, the pulse becoming regular, and of its usual rapidity, in about twenty minutes. The headache and nausea continued for nearly two hours. I afterwards passed a considerable quantity of clear urine, the reaction of which was acid, and of s. g. 1026, while that passed previously the same day, and that on the following day, was respectively 1019 and 1020. I could not, however, detect the smell of the oil either in the urine or the skin.

A man, employed in the operation of bagging or treading the dried hops into the pockets, told me that occasionally the smell of the hops made him so faint that he was obliged to go into the open air. He described his feelings as a faintness and dizziness; and seeing him on one of those occasions, I found his pulse slow and intermittent; and it is worth notice, that I have not heard of these effects being produced on those who are employed gathering the green hop in the hop-gardens. Besides the volatile oil, tannin, and extractive matter, lupuline contains a principle to which the name Humuline or Lupulite has been given; some writers have thought this to be the active ingredient of the hop, but I have never been able to satisfy myself that it possesses any sedative power. Its effects on the digestive organs are somewhat tonic. I have never observed from the humuline any of the symptoms produced by the oil. I have found much difficulty in obtaining sufficient humuline to make prolonged experiments; and my observations were made principally after the administration of lupuline, which had been deprived of its oil by distillation. I have never observed the effects of the oil produced. One day I took a strong decoction of lupuline, from which the oil had been distilled, and evaporated it. While this was progressing, I held my nostrils over the steam for half an hour. The smell was very different from that of the oil, being more pleasant, somewhat resembling that which is noticed in brewing. I observed no particular effect; certain it is that no nausea was produced. I have not succeeded in producing fatal effects on any of the lower animals by large doses, either of the oil or lupuline in substance. I administered as much as five drops of the former to a small terrier. I have, however, been told that a drove of ducks, a year or two ago, ate a large quantity of lupuline which had been swept from a hop-room into a poultry-yard, and that several of them died in consequence. It is also said that on hop-farms the lupuline has the property of making cows cast their calves, but I have never seen any parturient action from it on the human uterus.

With regard to the diseases in which lupuline may be given, I have prescribed it in a variety of cases with varying effects. It will be sufficient to give the general results of the cases, and not each one in detail. In diseases of the digestive organs, I have found it most useful in cases occurring in spirit drinkers, when there is tremulous tongue, loss of appetite, and excited manner. In pyrosis, it may advantageously be combined with bismuth, and in the dyspepsia of oxaluria, with the mineral acids. In some mild cases of gastralgia, it may be substituted with advantage for hydrocyanic acid. I have given it in several cases of articular rheumatism, without benefit, although Dr. Maton found its use beneficial in that disease. Three of five patients with sciatica expressed themselves relieved. In two cases of pleuritis it made no difference to the pain. In disorders of the nervous system, I have found its influence marked. One patient, (in a private lunatic asylum,) occasionally having epilectic seizures, and being in a highly nervous and excited state, begged for a dose of it nightly, after having once experienced its effects, as keeping away a "dreadful sensation," which prevented his sleeping, and was accompanied by incoherent muttering, of which the patient was himself unconscious. Two young ladies were seized with such pain as to cause them to scream loudly every few minutes. Dr. Evans, of Birmingham, who saw the patients in consultation, diagnosed the pain, which was seated in the abdomen, as hysterical; and after opium, morphia, camphor, and hyoscyamus had failed, and chloroform inhaled had only afforded partial benefit, prescribed lupuline, with positive and speedy relief to the symptoms.

A man aged forty-nine, a brass-founder, had suffered for two years from gradually increasing pain and tenderness on left side of the spinal column. When the pain was most acute, morphia internally gave but little relief, as also did the actual cautery applied over the spine; but he experienced decided benefit from repeated ten-grain doses of lupuline. A few weeks after, I heard that paralysis of the right leg had come on.

An elderly lady, the wife of a surgeon, was suffering from neuralgic pain in the back, of distressing severity of many days' continuance. She took henbane and tonics, used aconite and chloroform internally, without effect. Two or three doses of lupuline relieved her permanently. Four cases of chorea were not affected by its use. Lupuline, prescribed by Dr. Heslop, of Birmingham, for a lady with melancholia and loss of sleep, and in a similar case under Mr. Dufton, of Birmingham, had in both cases a decidedly satisfactory effect. Two cases of nymphomania seemed improved by its use; but as the patients were nearly idiotic, they were not very satisfactory subjects for observation. In some affections of the urino-genital organs, I can corroborate the favorable expressions of Zambaco and Hertsfelder. In five cases of slight spermatorrhœa and troublesome erections, it was of much service. One gentleman, who, though married and having children, was much troubled by nocturnal emissions, was free for many nights while taking lupuline. At the same time, I must in fairness state that Mr. L. Parker found it useless in a case in which he prescribed it. In several cases of painful erections in gonorrhœa, I have seen it serviceable; and it, as was observed in two cases of very chronic gleet, seemed to possess the power of abating the discharge; but in two cases of chordee, where there was decided curvature during erection, it proved useless. Dr. Heslop prescribed it nightly for a boy aged eight, who had had nocturnal enuresis since birth, and was admitted into the Queen's Hospital for modified variola, and after a week the disease was removed entirely.

Lupuline often removes the pain in back and chest accompanying simple leucorrhœa. I have also found it useful in one or two cases of ulceration of the os. It almost invariably relieves the pains in carcinoma uteri. I have found it do so in eleven cases, and in one of carcinomatous rectum: as also have Dr. Patterson, of Tiverton, in one case affecting the breast; Mr. Kite, of West Bromwich, in a similar case; and Dr. Bell Fletcher, of Birmingham, in several cases of cancer. In two cases of abortion, where the fœtus had been expelled, the separation of the placenta was not accelerated by its administration, though in each case this was speedily accomplished by a dose of ergot of rye. Lupuline completely relieved irritability of the bladder in two elderly females.

These are the conclusions I have arrived at with regard to the lupuline:—

1st. That it contains two distinct and different principles.

2d. That one of these, the oil, is a pure sedative and anodyne.

3d. That the other, which is probably humuline, has merely a tonic effect on the digestive organs.

4th. That the lupuline in substance may be given in very large doses, ten grains every half-hour, without producing dangerous effects.

5th. That the principal advantage it possesses over other anodynes in relieving the diseases above mentioned is, that, instead of diminishing the digestive power, it rather increases it. I would also remark, that its effects vary on different individuals, some requiring larger and more frequent doses than others, to produce the same results. In some the lupuline seems to lose its power after repeated administration.

I should remark, that the above notes being chiefly a record of my own observations, I have but slightly attended to the literature of the subject; at the same time, I am not unmindful of the merits of the justly celebrated men who have labored at the same subject as Drs. Maton, Ives, and Debout. Nor do I wish to take from them the credit of originality. With regard to its administration, I have used the lupuline in substance and in tincture, and I have tried a watery extract of lupuline, but found it inert, owing probably to the dissipation of the oil in the process of evaporation.

The tincture of lupuline I employ is of the proportion of one ounce and six drachms to half a pint of proof spirit. The ordinary dose I employed was one drachm, and the tincture is best made by percolation. If it is made by maceration, and the process continued long, or if the lupuline be old, the tincture becomes milky.

In general, I prefer giving the lupuline in substance, by placing ten grains on the tongue, and washing down with a little water; and this dose may be repeated every three or four hours if required, or the lupuline may be made into pills by manipulating it in a warm mortar.—*Edinburgh Medical Journal*, Feb. 1858.

